# Ethnic inequalities in the treatment and outcome of diabetes in three English Primary Care Trusts

**DOI:** 10.1186/1475-9276-6-8

**Published:** 2007-08-02

**Authors:** Michael A Soljak, Azeem Majeed, Joseph Eliahoo, Anne Dornhorst

**Affiliations:** 1 NHS London, Victory House, 170 Tottenham Court Road, London, W1T 7HA, UK; 2 Department of Primary Care and Social Medicine, Imperial College London,, London, SW7 2AZ, UK; 3 Statistical Advisory Service, Imperial College, South Kensington Campus, London, SW7 2AZ, UK; 4 Division of Investigative Science, Imperial College, Charing Cross Campus, St. Dunstan's Road, London, W6 8RP, UK

## Abstract

**Background:**

Although the prevalence of diabetes is three to five times higher in UK South Asians than Whites, there are no reports of the extent of ethnicity recording in routine general practice, and few population-based published studies of the association between ethnicity and quality of diabetes care and outcomes. We aimed to determine the association between ethnicity and healthcare factors in an English population.

**Methods:**

Data was obtained in 2002 on all 21,343 diabetic patients registered in 99% of all computerised general practitioner (GP) practices in three NW London Primary Care Trusts (PCTs), covering a total registered population of 720,000. Previously practices had been provided with training, data entry support and feedback. Treatment and outcome measures included drug treatment and blood pressure (BP), total cholesterol and haemoglobin A1c (HbA1c) levels.

**Results:**

Seventy per cent of diabetic patients had a valid ethnicity code. In the relatively older White population, we expected a smaller proportion with a normal BP, but BP differences between the groups were small and suggested poorer control in non-White ethnic groups. There were also significant differences between ethnic groups in the proportions of insulin-treated patients, with a smaller proportion of South Asians – 4.7% compared to 7.1% of Whites – receiving insulin, although the proportion with a satisfactory HbA1c was smaller- 25.6% compared to 37.9%.

**Conclusion:**

Recording the ethnicity of existing primary care patients is feasible, beginning with patients with established diseases such as diabetes. We have shown that the lower proportion of South Asian patients with good diabetes control, and who are receiving insulin, is at least partly due to poorer standards of care in South Asians, although biological and cultural factors could also contribute. This study highlights the need to capture ethnicity data in clinical trials and in routine care, to specifically investigate the reasons for these ethnic differences, and to consider more intensive management of diabetes and education about the disease in South Asian patients.

## Background

Some ethnic groups are at higher risk of developing diabetes. The 1999 Health Survey for England showed that, compared to the European population, diabetes is more common in South Asians (the UK Census category for people from the Indian Sub-Continent). Pakistanis and Bangladeshis were more than five times as likely to have diabetes, and in Indians the risk was three times higher [1]. Rates of diabetes among Black Caribbeans were also significantly higher than in the general population.

The same factors responsible for the higher prevalence of diabetes in some ethnic groups may also affect the course and outcomes of the disease. Relatively little is known about differences in the intermediate (metabolic) or longer term outcomes of diabetes in different ethnic groups, and whether the basis for any differences is mainly biological, cultural or related to inequalities in healthcare service delivery. The main reason is the lack of ethnicity information in epidemiological studies, clinical trials, and clinical databases.

A recent systematic review [2] reported ethnic differences in quality and intermediate outcomes of diabetes care to late 2004. Intermediate clinical outcomes were worse in Blacks and inclined to be worse in Hispanics. All the studies reviewed were from the US, and none quoted separate results for South Asians. Two more recent studies from the US used ethnicity information collected as part of clinical care to examine the processes and outcomes of diabetes care [3,4]. The second quoted ten other US studies of the effect of ethnicity on diabetes care and outcomes. However, because the US Census places all Asians and Pacific Islanders into a single group, none of these studies provided separate information on the South Asian ethnic group. Yet because pathophysiology may differ in this group, as evidenced by the higher prevalence, it is particularly important that both the quality of care and response to treatment are known.

A study of a London diabetic clinic, i.e. a population referred for specialist care, showed that South Asians with type 2 diabetes had poorer glycaemic, BP, and lipid control than white Europeans [5]. In another UK clinic population, glycaemic control was worse in South Asians than Europeans over time, with average deterioration 1.31% in Asians vs. 0.82% in Europeans [6]. Moreover, South Asians had significantly smaller improvements in BP and cholesterol over the follow-up period, in keeping with fewer prescriptions of anti-hypertensive agents and lipid-lowering agents. This suggests that healthcare delivery factors were at least partly responsible. A third study showed differences in improvements over time in intermediate outcomes, and poorer glycaemic control among patients of South Asian origin [7]. However these were all patients under the care of hospital diabetologists, and might not be representative of the entire diabetic population, which in the UK is largely managed in primary care.

Assessing treatment and outcomes of all diabetic patients registered for primary care is dependent on the extent of ethnicity coding as part of routine care delivery. The UK National Diabetes Audit, which involved 36 PCTs, attempted to analyse data by ethnicity, but this was impossible because recording of ethnicity was below 20 per cent in 32 of the 36 participating PCTs [8]. Several UK reports have attempted to fill this gap using ecological methods. To investigate the impact of ethnicity on diabetes prevalence, Goyder and Hammersley [9] collected data from 19 English practices using EMIS clinical information systems. Information was collected on a range of clinical data, but ethnicity could only be estimated by using data from the 1991 Census based on the resident population.

Also in the UK, Hippisley-Cox et al [10] examined the effect of deprivation and ethnicity on the achievement of quality of care indicators for patients with diabetes in 237 GP practices included in the QRESEARCH general practice database. Because of the lack of recorded ethnicity data, they too used deprivation scores and ethnicity proportions derived from the 2001 Census, both linked to the Output Areas associated with each patient's postcode, and showed that patients from high ethnic minority areas had poorer levels of recording of diabetes care. However use of Census data to impute patients' ethnicity introduces obvious inaccuracy. For example, in the UK patients are more likely to be registered with a GP of their own ethnic group, introducing another error in attributing ethnicity to practice populations.

The NHS Diabetes National Service Framework (NSF) [11] requires that, in addition to intensive glucose control i.e. an HbA1c less than 7.0%, hypertension and abnormal lipids should also be treated, with the aim of achieving the best possible level of overall metabolic control. In addition, the Quality & Outcomes Framework (QOF) of the new General Medical Services Contract for GPs incentivises the achievement of quality standards for diabetes [12].

We aimed to determine whether ethnicity and processes of care had independent effects on the NSF short-term outcome indicators in a multi-ethnic West London diabetic population, and if so, to determine which measures were affected. We used ethnicity information entered into practice computer systems by practice staff as part of routine care delivery to investigate the effect of ethnicity on short-term diabetes outcomes in a population with a large South Asian ethnic minority group.

## Methods

Data on diabetes treatment and intermediate outcomes was obtained from all computerised general practices in three North West London PCTs- Ealing, Hammersmith & Fulham and Hounslow- covering a total 2001 Census resident population of 678,000- although the population actually registered with a GP situated in the three PCTs was 720,000 (there is a high level of population mobility in London, with the result that at any one time some patients are registered with more than one practice). North West London is an ethnically and socio-economically diverse area, with large South Asian and significant Black African/Afro-Caribbean populations. Table 1 shows 2001 Census ethnicity data for residents of the three London Boroughs covered by the PCTs. Definitions of ethnicity were changed between the 1991 Census (which have continued to be used until recently by GP practices to define the ethnicity of their patients) and the 2001 Census. The biggest change in 2001 was the introduction of a new "Mixed" category, although the proportion who chose this category was small, and a change from the term "South Asian" to "Asian".

**Table 1:** Summarised 2001 Census ethnicity data for residents of the three London Boroughs

					**Mixed**				
						
**Borough of Residence**	**White N (%)**	**Asian N (%)**	**Black N (%)**	**Chinese or Other N (%)**	**White & Black Caribbean N (%)**	**White & Black African N (%)**	**White & Asian N (%)**	**Other N (%)**	**All people**
**Hammersmith & Fulham**	128,602 (77.8%)	7,333 (4.4%)	18,397 (11.1%)	4,610 (2.8%)	2,008 (1.2%)	1,033 (0.6%)	1,609 (1.0%)	1,650 (1.0%)	165,242
**Ealing**	176,741 (58.7%)	73,851 (24.5%)	26,456 (8.8%)	13,020 (4.3%)	3,022 (1.0%)	1,353 (0.4%)	3,629 (1.2%)	2,876 (1.0%)	300,948
**Hounslow**	137,754 (64.9%)	52,510 (24.7%)	9,237 (4.4%)	6,393 (3.0%)	1,382 (0.7%)	843 (0.4%)	2,407 (1.1%)	1,815 (0.9%)	212,341

**Total**	443,097 (65.3%)	133,694 (19.7%)	54,090 (8.0%)	24,023 (3.5%)	6,412 (0.9%)	3,229 (0.5%)	7,645 (1.1%)	6,341 (0.9%)	678,531

Source: 2001 Census

In November 1997 a chronic disease register (CDR) was established to offer support to all GP practices in the PCTs. The CDR supported patient care by downloading pseudo-anonymised (minus name and address) Read-coded data from practice systems every quarter using floppy discs containing Morbidity QUery Information Export SynTax (MIQUEST) queries, to produce quarterly patient- and practice-level reports which were re-linked with patient details by the practices. MIQUEST queries will capture all correctly-coded information.

At the same time guidance and training on ethnic coding using 1991 Census codes was disseminated. Feedback to practices included reports on the completeness of ethnicity and care standards data to encourage improvement. This feedback, training and some external clerical data entry support (in part for ethnicity coding) was most intensive and widespread over the period 2000–2002. Ethnicity data entry was carried out mainly by GPs, practice nurses and well-trained clerical staff. Some practices used patient questionnaires provided to them.

The period studied predates the commencement of the Quality & Outcomes Framework of the new GP Contract by almost two years, and no financial incentives were offered to practices. There were no major efforts to improve ascertainment or case-finding during the study period. The 2004–5 UK National Diabetes Audit estimated that 81 percent of the people predicted (utilising the PBS phase 2 diabetes population prevalence model) to have diabetes are actually recorded as having diabetes at GP practices [], and we would expect this proportion to be registered on the CDR.

Soon after their formation, the seven Primary Care Groups in the study area, the predecessors of the PCTs, set an objective of 100 percent practice participation in the CDR by October 2002. The number of practices included here is 173 of 178 who were capable/computerised. As the number of practices participating increased, there was an increase in the number of diabetic patients identified, from 13,686 in January 2001 to 21,343 by August 2002.

Both Type 1 and Type 2 diabetes has been included in the analysis, as have type 1 patients who are children. Gestational diabetes was not excluded or analysed separately as there was not a reliable means of doing so. The cut-off points chosen for the intermediate outcomes of BP, total cholesterol and HbA1c levels are derived from the 2002 NICE diabetes guidelines [13,14]. The 2002 NICE guidance recommends a range of 6.5–7.5% for HbA1c and the diabetes NSF refers to an optimal level of 7.0%. The guidance recommends achieving a BP of 140/80, so we used a BP cutoff of < 145 systolic. The most recent values in the last five years were analysed.

### Statistical methods

To test for the differences between ethnic groups, the Kruskal-Wallis test was used for age and body mass index (BMI) because they were non-normally distributed, and the Chi-square test for categorical variables. The p-value is for a test including the extra 'not recorded' category for ethnicity and for the variable of interest, if categorical i.e. the Chi-square test excludes the missing value category.

## Results

A total of 21,343 patients were identified with a diagnosis of either Type 1 or Type 2 diabetes. Table 2 shows the age structure of the resident population in the three Boroughs. Compared to the United Kingdom, they contain a higher proportion of working age population and a lower proportion of the older population.

**Table 2:** Summarised mid-2002 population estimates in selected age groups for residents of the three London Boroughs and the United Kingdom, in thousands

**Area/Borough**	**All Ages**	**Children 0–15**	**Working Age 16–64M/59F**	**65M/60F and over**
United Kingdom	59,321.7	11,783.4 (19.9%)	36,622.0 (61.7%)	10,916.3 (18.4%)
Hammersmith and Fulham	172.7	27.8 (16.1%)	124.4 (72.0%)	20.5 (11.9%)
Ealing	307.8	58.9 (19.1%)	208.3 (67.7%)	40.6 (13.2%)
Hounslow	215.4	43.0 (20.0%)	144.1 (66.9%)	28.3 (13.1%)

Source: ONS Projections From 2001 Census

Table 3 shows the ethnic group classification (using Read codes for 1991 Census categories) of the diabetic population recorded in GP systems in August 2002. Seventy per cent had a valid ethnic code. The corresponding figure in March 2001 was 60 percent. However, although there is a Read code for "Other Ethnic Groups", there is no definition in the 1991 Census for the composition of this group, so that only 64 per cent had usable codes. As the data was anonymised for confidentiality reasons, we were unable to employ software which uses surnames to assign ethnicity. Unfortunately ethnicity recording in the entire GP registered population was too low to be used to assess prevalence in each ethnic group. However while only 21 percent of the resident population is Asian or White & Asian, 41 percent of the diabetic population with ethnicity coded was South Asian.

**Table 3:** Ethnic group of diabetic population recorded in GP systems

**ETHNICITY**	**Freq**	**Percent**
**White**	**6,229**	**29.2%**
Indian	4,478	21.0%
Bangladeshi	118	0.6%
Pakistani	913	4.3%
**South Asian**	**5,509**	**25.8%**
Black African	413	1.9%
Black Afro-Caribbean	1,160	5.4%
Black, Other	133	0.6%
**Black African/Afro-Caribbean**	**1,706**	**8.0%**
Chinese	121	0.6%
Other Ethnic Groups	1,355	6.3%
Uncoded	6,423	30.1%

**TOTAL**	**21,343**	**100.0%**

Table 4 shows the descriptive, process or treatment measures and intermediate outcome measures by ethnic group. The White diabetes patient population was substantially older, in line with the population age structure. There were substantially more female patients in the Black African/Afro-Caribbean group. There were significantly less smokers in the South Asian population. Patients with ethnicity not recorded have poorer quality of recording overall, with a substantially higher percentage of missing values across all variables. They also have poorer outcomes for blood pressure control, total cholesterol and HbA1c. There is insufficient information in our dataset e.g. numbers of attendances to investigate the reasons for these findings further.

**Table 4:** Descriptive, treatment and intermediate outcome measures by ethnic group

	**Ethnic Group N (%)**					
	**White**	**South Asian**	**Black African/Afro-Caribbean**	**Other**	**Not recorded**	p-value*

**Descriptive Measures**						

**Mean Age**	65.4	59.7	62.8	58.9	61.8	p < 0.001
**Median (IQR)**	(54.2–74.4)	(50.2–67.6)	(52.3–69.4)	(49.4–67.9)	(49.2–71.7)	

**Sex N (%)**						
**Male**	3463 (55.4)	3140 (56.6)	778 (45.9)	750 (53.7)	3507 (54.4)	p < 0.001
**Female**	2775 (44.4)	2381 (42.9)	913 (53.8)	638 (45.7)	2900 (45.0)	
**Not recorded**	15 (0.2)	30 (0.5)	6 (0.4)	9 (0.6)	38 (0.6)	

**Mean BMI **	28.7	27	28.5	27.3	27.3	p < 0.001
**Median(IQR)**	(25.2–33.0)	(24.3–30.4)	(25.5–32.2)	(24.6–31.1)	(24.2–31.2)	
**Not recorded N(%)**	870(13.9)	495(8.9)	233 (13.73)	195 (14.0)	2361(36.6)	

**Smoker N(%)**	1152 (18.4)	389 (7.0)	190 (11.2)	179 (12.8)	822 (12.8)	p < 0.001
**Not recorded**	281 (4.5)	430 (7.8)	82 (4.8)	94 (6.7)	1508 (23.4)	p < 0.001

**Treatment Measures**						
**On insulin N (%)**	443 (7.1)	263 (4.7)	126 (7.4)	80 (5.7)	592 (9.2)	p < 0.001
**On statins N (%)**	1860 (29.8)	1744 (31.4)	425 (25.0)	399 (28.6)	1405 (21.8)	p < 0.001

**Intermediate Outcome Measures**						
**HbA1c < 7.4 N (%)**	2368 (37.9)	1423 (25.6)	540 (31.8)	438 (31.4)	1354 (21.0)	p < 0.001
**Not recorded**	1100 (17.6)	1377 (24.8)	313 (18.4)	320 (22.9)	3248 (50.4)	
**BP < = 145 N (%)**	4094 (65.5)	3711 (66.9)	1095 (64.5)	995 (71.2)	3604 (55.9)	p < 0.001
**Not recorded**	119 (1.9)	57 (1.0)	21 (1.2)	32 (2.3)	1035 (16.1)	
**Cholesterol < = 5 N(%)**	2537 (40.6)	2275 (41.0)	711 (41.9)	580 (41.5)	1609 (25.0)	0.038
**Not recorded**	1056 (16.9)	1154 (20.8)	265 (15.6)	226 (16.2)	2953 (45.8)	p < 0.001

* note that the Chi-square tests for care process and intermediate outcome measures exclude the missing value category

Total cholesterol, HbA1c, and insulin prescription were recorded less often for South Asians, and the latter two were also recorded less often for the Other ethnic group. In that there is no Census definition, the Other ethnic group could be seen as a proxy for relatively poor quality of data recording.

There were significant differences in the proportion of insulin-treated patients, with a smaller proportion of South Asians receiving insulin, although the proportion of South Asians with a satisfactory HbA1c is smaller. We therefore expected higher insulin prescribing. On the other hand, prescribing of statins is significantly higher in South Asians, although there is no significant difference in the proportion of patients with a total cholesterol less than 5 mmol/l. A significantly higher proportion of Black Africans/Afro-Caribbeans have an abnormally high systolic blood pressure.

## Discussion

By 2002, about 70 per cent of diabetic patients in NW London had an ethnic group recorded in their electronic care record, although six per cent of these had a category of "Other". This was a 10 percent improvement over the prior 15 months. Although we know that patients with a chronic disease are more likely to have ethnicity recorded, this result was achieved with only moderate levels of training, support and encouragement to GP practices, and before ethnicity recording of new patients was incentivised through the GP Contract Quality & Outcomes Framework. We would therefore expect further improvements to have occurred recently.

There is a high level of awareness of the importance of ethnicity as a disease risk factor, and many practices appear to be using ethnicity as an indicator of risk for clinical management purposes already. It is clearly feasible to extend the recording of ethnicity at the time of new patient registration to all patients with QOF diseases across the UK as a next step.

Some classification bias could have occurred in our study if patients from ethnic minorities were more or less likely to have their ethnic group coded. However the relatively high level of coding reduces error from this source. Our findings suggest that lack of recording is a proxy for overall poor quality of recording and of clinical care.

A previous UK study of prescribing data showed that patients in practices with a greater South Asian population are less likely to be prescribed lipid-lowering drugs [15], and another that GP prescribing rates in some PCTs were negatively associated with proxies of healthcare need based on patient age, ethnicity, levels of deprivation, and standardised mortality ratios (SMRs) for CHD [16]. A subsequent analysis of data from the Health Survey for England showed an association between deprivation and lower prescribing, but not ethnicity, highlighting the well-known interaction between these two variables [17].

The Scottish hospital clinic study (which included only 1700 patients) showed South Asians had fewer prescriptions of anti-hypertensive agents and lipid-lowering agents and significantly smaller improvements in blood pressure and cholesterol [6]. In contrast our data shows similar levels of statin prescribing in all patients except in Black Africans/Afro-Caribbeans, in whom it is significantly lower, while proportions with a normal cholesterol are not significantly different in all ethnic groups. Since the local White population is significantly older, we expected a smaller proportion to have a normal blood pressure, but differences between the groups with ethnicity coded is small, suggesting poorer outcomes in minority ethnic groups. Overall there does not appear to be a consistent picture for these processes and outcomes in diabetic patients.

For blood glucose control, an overall comparison with a Swedish population-based study suggests that, in the outcome of care is somewhat poorer in NW London [18]. In the Swedish population, only 40 per cent had an HbA1c greater than 7.5 per cent, while in NW London the proportion was 59 per cent of those with HbA1c recorded, for all ethnic groups. We consider that these findings are probably typical of similar UK populations.

A study from North Eastern USA [3] showed significantly higher HbA1c in Black women but not Black men with established diabetes, although a significantly higher proportion were insulin-treated. The proportion of UK Afro-Caribbeans who had achieved the target HbA1c fell between those of the other two ethnic groups, and the proportion who were insulin-treated was similar to Whites. In the other US study from the TRIAD group [4] mean HbA1c was higher in the ethnic minority patients. It was not possible to compare insulin treatment from the data provided in this study, or to analyse South Asians separately.

Although the proportion of South Asian patients with good diabetes control is significantly lower in our population, a lower proportion are receiving insulin. This could be due to poorer patient concordance with treatment (or GPs' perceptions of concordance) [19], or other undetected differences in practices' quality of care, such as less intensive management because of outdated knowledge. The low proportion of GP-based diabetic patients on insulin was probably typical for NW London in 2002. Insulin use in secondary care for type 2 diabetes has risen in the last 5–10 years, but in primary care the initiation of insulin has risen more slowly, and has only accelerated in the last 2 years following the use of once daily long acting insulin with the simultaneous use of oral agents. Primary care services are free in the UK, the vast majority of the population is registered with a GP, and most receive free prescriptions. However while deprived UK populations have higher utilisation rates for unscheduled care, this is not the case for preventive primary care. This is probably due to a mixture of patient-and provider-related factors such as lower health literacy, and poorer care standards in deprived areas. For example, some South Asians patients with DM have ambivalent attitudes to diabetic treatments, and so may be more reluctant to start insulin [19]. We consider that this demonstrates poorer standards of care in South Asians, but that institutional racism is unlikely to be a major cause, as many South Asian patients are registered with GPs from their own ethnic group.

It is also possible that South Asians respond less well to anti-diabetic agents. Levels of glucose intolerance, central obesity (as measured by waist to hip ratio), fasting triglyceride, and insulin are uniformly elevated in South Asians compared to Europeans [20]. These factors are key features of the insulin resistance syndrome, and probably explain the higher prevalence of diabetes in this group. However there are no previous population-based reports documenting ethnic differences in metabolic responsiveness to anti-diabetic agents. This highlights the need to capture ethnicity data in clinical trials as well as in routine care, and to specifically investigate the reasons for these ethnic differences by monitoring outcomes in populations with the same standards of care.

## Conclusion

We have reported for the first time on the process and outcomes of diabetes care in a population-based study using ethnicity data captured during routine primary care. About 70% of patients in NW London have valid ethnicity codes. Diabetes control was significantly worse in South Asians, but a smaller proportion of this group were insulin treated. We have therefore confirmed earlier reports in clinic populations in a much larger, population-based group that some outcomes of care- in particular blood glucose control- are worse in some UK ethnic minority groups. We have shown that this is at least partly due to poorer standards of care in South Asians, although biological factors could also contribute. Further studies are required to determine the extent to which provider or patient factors- behavioural or biological- are responsible.

## Competing interests

The author(s) declare that they have no competing interests.

## Authors' contributions

MS managed the original project from which the data was obtained and wrote the article text. AM provided ongoing advice on analysis and drafting. JE carried out the statistical analysis. AD commented on final drafts. We acknowledge the help of Phil Kirby and Dominic Clarke, who extracted the data. All authors have read and approved the final manuscript.
